# A comparison of the ability of the National Early Warning Score and the National Early Warning Score 2 to identify patients at risk of in-hospital mortality: A multi-centre database study

**DOI:** 10.1016/j.resuscitation.2018.09.026

**Published:** 2019-01

**Authors:** Marco A.F. Pimentel, Oliver C. Redfern, Stephen Gerry, Gary S. Collins, James Malycha, David Prytherch, Paul E. Schmidt, Gary B. Smith, Peter J. Watkinson

**Affiliations:** a Institute of Biomedical Engineering, Department of Engineering Science, University of Oxford, Oxford, UK; b Centre for Healthcare Modelling and Informatics, University of Portsmouth, Portsmouth, UK; c Centre for Statistics in Medicine, Nuffield Department of Orthopaedics, Rheumatology and Musculoskeletal Sciences, Botnar Research Centre, University of Oxford, Oxford, UK; d Nuffield Department of Clinical Neurosciences, University of Oxford, Oxford, UK; e Department of Medicine, Portsmouth Hospitals NHS Trust, Portsmouth, UK; f Faculty of Health and Social Sciences, Bournemouth University, Bournemouth, UK

**Keywords:** Physiological monitoring, Early warning score, Vital signs, COPD

## Abstract

**Aims:**

To compare the ability of the National Early Warning Score (NEWS) and the National Early Warning Score 2 (NEWS2) to identify patients at risk of in-hospital mortality and other adverse outcomes.

**Methods:**

We undertook a multi-centre retrospective observational study at five acute hospitals from two UK NHS Trusts. Data were obtained from completed adult admissions who were not fit enough to be discharged alive on the day of admission. Diagnostic coding and oxygen prescriptions were used to identify patients with type II respiratory failure (T2RF). The primary outcome was in-hospital mortality within 24 h of a vital signs observation. Secondary outcomes included unanticipated intensive care unit admission or cardiac arrest within 24 h of a vital signs observation. Discrimination was assessed using the c-statistic.

**Results:**

Among 251,266 adult admissions, 48,898 were identified to be at risk of T2RF by diagnostic coding. In this group, NEWS2 showed statistically significant lower discrimination (c-statistic, 95% CI) for identifying in-hospital mortality within 24 h (0.860, 0.857–0.864) than NEWS (0.881, 0.878-0.884). For 1394 admissions with documented T2RF, discrimination was similar for both systems: NEWS2 (0.841, 0.827-0.855), NEWS (0.862, 0.848–0.875). For all secondary endpoints, NEWS2 showed no improvements in discrimination.

**Conclusions:**

NEWS2 modifications to NEWS do not improve discrimination of adverse outcomes in patients with documented T2RF and decrease discrimination in patients at risk of T2RF. Further evaluation of the relationship between SpO_2_ values, oxygen therapy and risk should be investigated further before wide-scale adoption of NEWS2.

## Introduction

Vital signs based aggregate early warning score (EWS) systems, which assign weights to each vital sign according to the deviation from assumed normal values, are recommended for routine use in UK hospitals^1^, ^2^. In 2012, the Royal College of Physicians of London (RCPL) published a proposed National EWS (NEWS)^3^, which has now undergone extensive validation^4^, ^5^, ^6^. In NEWS, oxygen saturations (SpO_2_) receive increasing weights for values of 95% or less, and oxygen therapy receives a flat weight. However, guidance for the management of patients with type II respiratory failure (T2RF)^7^, ^8^, and those deemed at risk of T2RF before blood gas analysis^7^, suggests lower SpO_2_ values (88–92%) should be targeted. Consequently, it is suggested that the NEWS SpO_2_ weighting system is inappropriate for patients with/at risk of T2RF^9^, ^10^, ^11^. Some authors suggest that this weighting risks inappropriate oxygen therapy for these patients, with potential deleterious consequences^9^, ^10^.

In December 2017, the RCPL published an update to NEWS - the National Early Warning Score 2 (NEWS2)^12^ - which includes several modifications to the NEWS vital sign weightings. To account for concerns about NEWS and T2RF, NEWS2 includes a new SpO_2_ scoring scale for patients with/at risk of T2RF. This scale, termed *SpO_2_ scale 2* assigns weights at lower SpO_2_ thresholds than NEWS and combines these lower thresholds with weights for the use of supplemental oxygen at higher SpO_2_ levels, reflecting the concern of hyperoxia-induced hypercapnic respiratory failure^12^ (see appendix A1). Although the derivation of these thresholds is not presented, and NEWS2 is as yet unvalidated, NHS England has endorsed NEWS2′s use in acute and ambulance settings^13^, and is considering the use of the Commissioning for Quality and Innovation (CQUIN) payment system^14^, ^15^ to encourage organisations to implement NEWS2 by March 2019.

In this study, we used a large multi-centre dataset of vital signs to compare retrospectively the performance of NEWS2 and NEWS. We studied the performance of NEWS and NEWS2 in three risk groups: those with documented T2RF; those at risk of T2RF; and patients in neither of these groups.

## Methods

The database for this study was created with Health Research Authority (reference: 16/SC/0264 and 08/02/1394) approval. The study protocol is available online^16^; we follow the TRIPOD statement for reporting^17^.

### Source of data

A database of vital sign observations was collated from adult (aged at least 16 years) acute admissions to the Oxford University Hospitals (OUH) group and Portsmouth Hospitals NHS Trust (PH) as part of the Hospital Alerting Via Electronic Noticeboard (HAVEN) dataset^18^. Clinical staff recorded patients’ vital signs at the bedside using the System for Electronic Notification and Documentation (SEND, Drayson Health, www.draysonhealth.com)^19^ in OUH and VitalPAC^™^ (System C Healthcare, www.systemc.com) in PH^20^. The following data were recorded: date and time of observation (automatically by SEND/VitalPAC^™^); heart rate, systolic blood pressure, respiratory rate, body temperature, neurological status using the Alert-Voice-Pain-Unresponsive (AVPU) scale, SpO_2_; and the patient’s inspired gas (air or supplemental oxygen) at the time of SpO_2_ measurement. The HAVEN database also contains administrative and patient demographic information, and information about the occurrence and timing of cardiac arrest, unanticipated intensive care unit (ICU) admission and hospital discharge status (dead/alive). Prescription data from the electronic patient record is also available within the database for OUH admissions.

### Study sites

The study took place at five hospitals – the four hospitals in the OUH group [The John Radcliffe Hospital (large university hospital), The Horton General Hospital (small district general hospital), The Churchill hospital (large university cancer centre) and The Nuffield Orthopaedic Hospital] and a single large district general hospital, PH.

### Participants

All completed adult admissions to the four hospitals comprising the OUH group (January-December 2016) and to PH (January 2012 - December 2016) with at least one complete set of vital signs observations recorded electronically were considered. These study periods represent times of full deployment of electronic vital signs documentation in these hospitals. Patients discharged alive from the hospital before midnight on the day of admission and those with no vital signs recorded in the 24 h prior to discharge (as a proxy for patients on end-of-life pathways) were excluded from the analysis. For the main analysis, we combined admissions from all hospitals, but we also analysed data from each hospital trust separately (see appendix A3).

### Early warning scores (see appendix A1)

The NEWS2 adjustment for patients with/at risk of T2RF differs from NEWS in the assignment of weights to measured SpO_2_ (NEWS weights SpO_2_ values below 96%; NEWS2 below 88%). Additionally, for patients with/at risk of T2RF, NEWS2 assigns weights for SpO_2_ values above 92% when receiving oxygen.

### Outcome

The primary outcome was in-hospital death within 24 h of an observation set, in line with previous studies^21^, ^22^. Secondary outcomes include cardiac arrest, unanticipated ICU admission, and either cardiac arrest, unanticipated ICU admission, or death within 24 h of an observation set. We present the results for all secondary outcomes, flagging those where insufficient outcomes exist (< 100), due to sample size, as recommended in the TRIPOD guidelines^17^. All outcomes were obtained retrospectively from different clinical information systems, including the hospitals’ patient administration systems, the ICU clinical information systems, and the hospitals’ National Cardiac Arrest Audit (https://ncaa.icnarc.org) databases.

### Predictors

Vital sign sets (see above) were recorded using SEND/VitalPAC^™^. Where the patient’s conscious level had been assessed only using the Glasgow Coma Scale (GCS), we converted GCS to an AVPU equivalent^21^. Vital signs were then assigned weights for NEWS and NEWS2 scores (see appendix A1). The sum of the weights (aggregate score) results in the NEWS and NEWS2 value for each observation set. SEND (OUH) uses a modified EWS, CEWS^23^, which assigns increasing weights to SpO_2_ values less than 94% and does not weight SpO_2_ values of 94% or above. Clinical staff entering vital signs data were therefore unaware of NEWS or NEWS2 scores. VitalPAC^™^ (PH) uses NEWS. To allow comparison with published analyses of NEWS^22^, ^24^, and in line with previous vital-signs-based EWS research^25^, ^26^, ^27^, ^28^, each vital sign set was analysed as independently associated with the outcome.

### Missing data

For the analysis, we considered complete observation sets (i.e., sets with measurements of all vital signs), in line with previous NEWS studies^22^, ^24^. The SEND system allows recording of incomplete vital sign sets, which is discouraged in the VitalPAC^™^ system. We did an *a priori* sub-analysis in which we used *multiple imputation*, a general-purpose and widely used approach to missing values^29^ which only occurred in the OUH dataset.

### Statistical analysis

Performance of NEWS and NEWS2 was assessed by discrimination using receiver operating characteristic (ROC) curve analysis (calibration was not assessed, as the EWS systems do not give estimates of absolute risk). We also assessed the effect of suggested thresholds for patient review (aggregate NEWS/NEWS2 scores of 5 or above, or 7 or above^12^) by reporting sensitivity, specificity and positive predictive values. We also show SpO_2_ distributions for three different risk groups (see below). All analysis was performed using the R statistical software (v3.4.4)^30^ and ROC curves were calculated using the pROC package^31^. Differences in the area under the ROC curve (AUROC), or c-statistic, between NEWS and NEWS2 were compared using bootstrapping (2000 samples)^31^. We did *post-hoc* sub-analyses of performance by institution (in light of the different patient numbers contributed). We also performed *post-hoc* efficiency curve analysis (as we were unable to conduct decision curve analysis as estimates of risk for a given score are not available).

### Risk groups

After exclusion criteria were applied, we categorised each admission according to the following risk groups:

1 Patients with recorded T2RF, identified using the *Adult Oxygen Prescription* form of the current admission (OUH only).
2 Patients at risk of T2RF, identified using the International Statistical Classification of Diseases and Related Health Problems-10 (ICD-10) classification for their concurrent or prior admission, with either of the following groups of diagnosis codes:
  a J40-J44 (typically, 88% coded as J44) - patients with Chronic Obstructive Pulmonary Disease (COPD); or
  b J47 – patients with Bronchiectasis; or
  c E84 – patients with Cystic Fibrosis; or
  d E66 – patients with obesity and/or obesity hypoventilation syndrome; or
  e G12, G47.3, G70-G71, M95.4, or Q67.8, with J96 – patients with respiratory failure (J96) and one of the following conditions: spinal muscular atrophy and other motor neuron disease (G12), sleep apnoea (G47.3), myasthenia gravis and other myoneural disorders (G70), muscular dystrophies (G71), acquired deformity of chest and rib (M95.4), or other congenital deformities of chest (Q67.8).
3 Patients not at risk of T2RF, i.e., not in groups 1 or 2 above.
  We report the performance metrics of each scoring system for each of these risk groups. We report the results of the SpO_2_ scale 2 of NEWS2 in the third risk group (patients not at risk of T2RF) to demonstrate the effect of erroneous use of the scale in this population.

### Development versus evaluation datasets

NEWS was originally developed using a dataset with admissions to PH’s Medical Assessment Unit (MAU) [22]. The NEWS2 report does not identify a development dataset for NEWS2^12^. The study evaluation database (HAVEN) includes data from all admissions to OUH and pH for the periods stated above. Vital sign data for all sites are present from hospital admission to hospital discharge/death. NEWS2 is recommended for use in all the included settings.

## Results

### Descriptive statistics

**Fig. 1:**
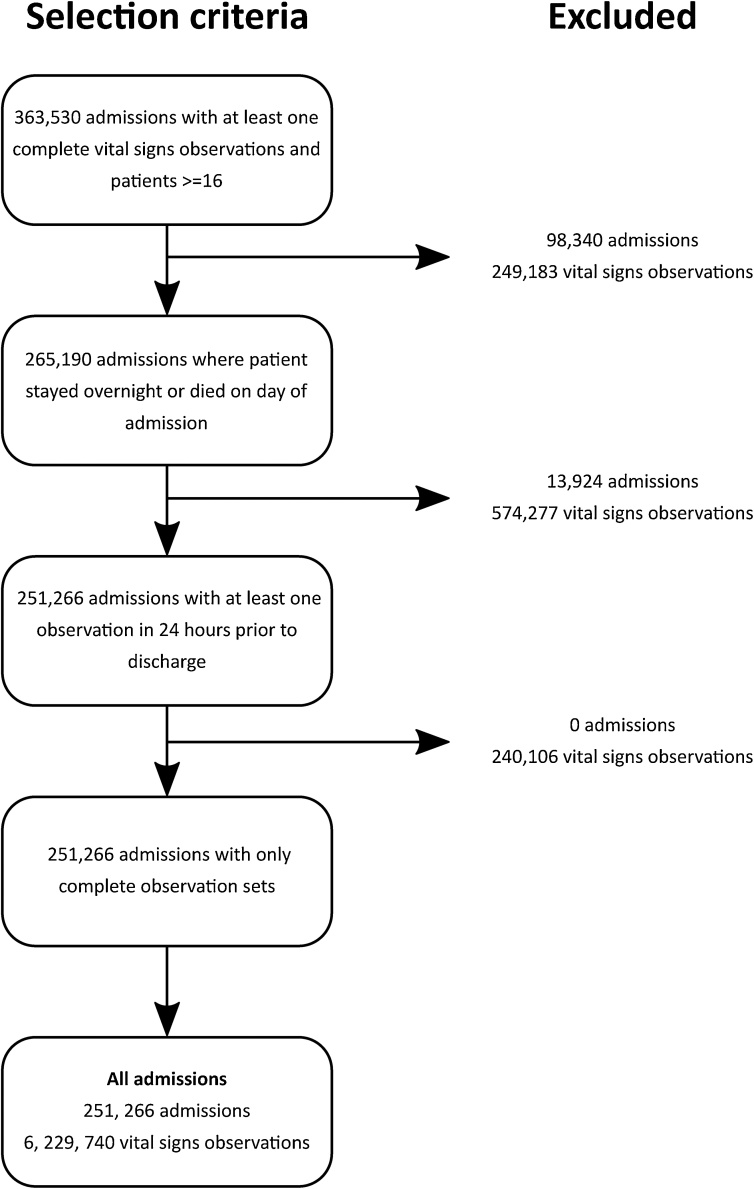
Flowchart showing application of exclusion criteria for obtaining the admissions included in the analysis.

A total of 251,266 distinct admissions were included. Fig. 1 shows the application of inclusion/exclusion criteria, resulting in the final cohort of admissions. All patients in the final dataset had at least one complete vital sign set. A total of 48,898 admissions were associated with patients at risk of T2RF, and 1394 with patients with documented T2RF (80.3% of whom also belong to the group of patients at risk of T2RF). Table 1 summarises the admission demographic descriptors and other clinical information for the three risk groups. Patients in risk groups 1 (documented T2RF) and 2 (at risk of T2RF) both had higher mortality rates (and rates of other adverse outcomes) when compared to patients who were *not* at risk (i.e. risk group 3).

**Table 1:** Demographic descriptors for admissions included in each risk group. T2RF denotes Type II Respiratory Failure.

	Documented T2RF	At risk T2RF	Not at risk T2RF	All
Number of admissions	1394	48,898	202,094	251,266
Males, N (%)	696 (49.9)	23,569 (48.2)	95,736 (47.4)	119,433 (47.5)
Age (years), median (IQR)	75 (67-83)	72 (60-80)	66 (47-80)	68 (50-80)
Length of stay (days), median (IQR)	6.7 (3.1-14)	4.0 (1.8-9)	2.8 (1.3-6.8)	3.0 (1.3-7.1)
Charlson Comorbidity Index^a^, median (IQR)	7 (4-16)	4 (0-14)	0 (0-8)	0 (0-10)
Elective admissions^a^, N (%)	104 (7.5)	9351 (19.1)	49,978 (24.7)	59,374 (23.6)
Surgical admissions^a^, N (%)	229 (16.4)	14,833 (30.3)	89,427 (44.3)	104,338 (41.5)

Ethnic category, N (%)				
Asian or Asian British	23 (1.6)	281 (0.6)	1850 (0.9)	2136 (0.9)
Black or Black British	1 (0.1)	116 (0.2)	1031 (0.5)	1147 (0.5)
Mixed	9 (0.6)	117 (0.2)	710 (0.4)	828 (0.3)
Other Ethnic Groups	126 (9.0)	4317 (8.8)	29,585 (14.6)	33,936 (13.5)
Not disclosed	5 (0.4)	142 (0.3)	957 (0.5)	1102 (0.4)
White	1230 (88.2)	43,925 (89.8)	167,961 (83.1)	212,117 (84.4)

Primary outcome, N (%)				
In-hospital mortality	159 (11.4)	2220 (4.5)	4606 (2.3)	6871 (2.7)

Secondary outcome, N (%)				
Unanticipated ICU admission	45 (3.2)	575 (1.2)	1704 (0.8)	2289 (0.9)
Cardiac arrest	18 (1.3)	288 (0.6)	628 (0.3)	920 (0.4)
Number of vital sign sets	61,340	1,466,420	4,751,323	6,229,740

a The Charlson Comorbidity Index, and definitions of surgical specialties and elective admissions were determined according to the methodology and specification provided by NHS Digital (Charlson Comorbidity Index guidelines are available at https://beta.digital.nhs.uk/publications/ci-hub/summary-hospital-level-mortality-indicator-shmi).

**Fig. 2:**
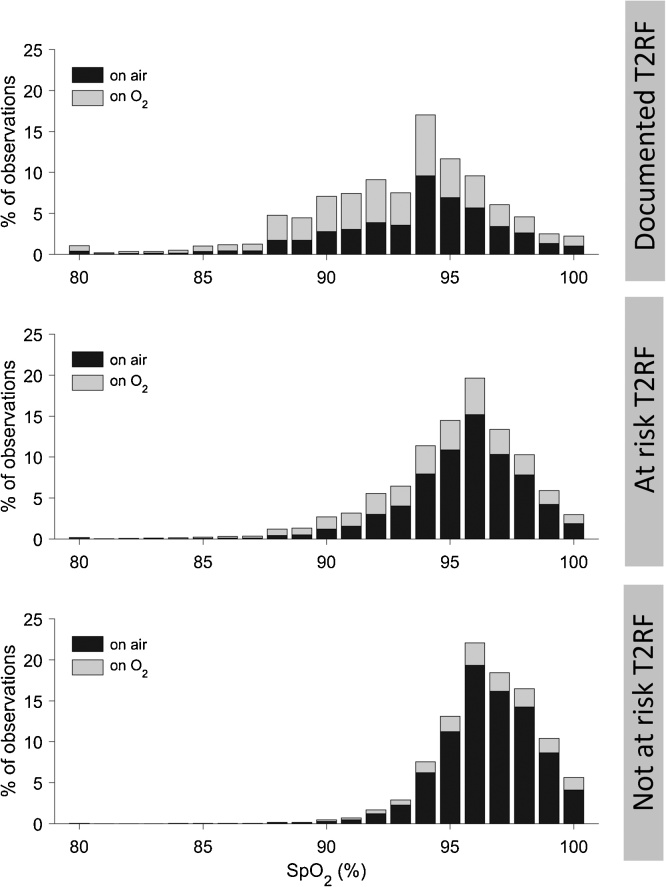
Representation of the normalised histograms of oxygen saturation (SpO_2_) recorded for each of the risk groups. For each bar, the relative proportion of measurements performed while patients were on oxygen (O_2_) or on air is shown.

The distribution of SpO_2_ values for patients with documented T2RF is bell-shaped, whereas that for the group of patients who are not at risk was right skewed (Fig. 2). In patients with documented T2RF, 77.4% of admissions had at least one recorded SpO_2_ measurement above 92% on room air, compared with 98.7% in the non-risk group (Fig. 2).

### Performance of early warning scores

**Fig. 3:**
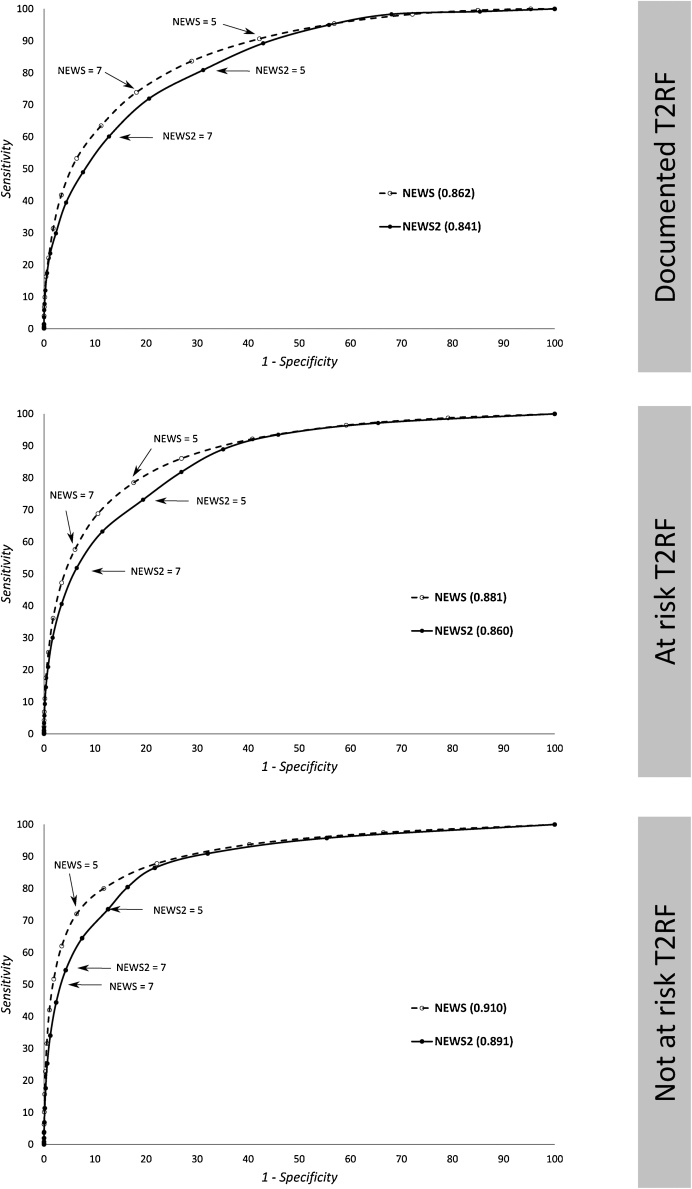
Receiver operating characteristic (ROC) curve for NEWS and NEWS2 (with scale2), for discriminating vital signs observations followed by in-hospital death within the following 24 h for the three risk groups (from top to bottom): admissions with documented type II respiratory failure (T2RF), admissions at risk of T2RF, and admissions not at risk of T2RF. Sensitivity and 1–Specificity are shown in %.

Performance metrics for the three risk groups for in-hospital death are presented in Table 2, and the corresponding ROC curves are represented in Fig. 3. Those for the secondary outcomes are shown in Table 3.

**Table 2:** Performance metrics of the two scoring systems (NEWS and NEWS2) for predicting the primary outcome in the three risk groups, which include the area under the receiver operating characteristics curve (AUROC), with 95% confidence interval (CI), and sensitivity, specificity and positive predictive value values at a threshold of 5 and 7. The fourth column (NEWS – NEWS2) indicates the mean difference (95% CI) between the AUROCs of NEWS and NEWS2. T2RF denotes Type II Respiratory Failure.

	NEWS	NEWS2	NEWS – NEWS2
Documented T2RF			
AUROC (95% CI)	0.862 (0.848 - 0.875)	0.841 (0.827 - 0.855)	0.021 (0.012 - 0.030)†
Score ≥ 5 / Score ≥ 7			
Sensitivity	90.7 / 73.9	80.9 / 60.1	
Specificity	57.8 / 88.8	68.8 / 87.3	
Positive predictive value	2.5 / 4.6	3.0 / 5.3	

At risk T2RF			
AUROC (95% CI)	0.881 (0.878 - 0.884)	0.860 (0.857 - 0.864)	0.021 (0.019 - 0.023)†
Score ≥ 5 / Score ≥ 7			
Sensitivity	78.5 / 57.6	73.2 / 51.8	
Specificity	82.4 / 93.9	80.6 / 83.6	
Positive predictive value	3.2 / 6.6	2.7 / 5.7	

Not at risk T2RF			
AUROC (95% CI)	0.910 (0.907 - 0.912)	0.891 (0.889 - 0.893)	0.019 (0.018 - 0.020)†
Score ≥ 5 / Score ≥ 7			
Sensitivity	72.0 / 51.7	73.5 / 54.5	
Specificity	93.6 / 98.1	87.4 / 95.7	
Positive predictive value	5.0 / 11.2	2.7 / 5.7	

† Denotes significant difference in AUROC (p < 0.05).

**Table 3:** Performance metrics of the two scoring systems (NEWS and NEWS2) for predicting the secondary outcomes in the three risk groups: area under the receiver operating characteristics curve (AUROC), with 95% confidence interval (CI). NEWS – NEWS2 indicates the mean difference (95% CI) between the AUROCs of NEWS and NEWS2. T2RF denotes Type II Respiratory Failure.

	Documented T2RF	At risk T2RF	Not at risk T2RF
Unanticipated ICU admission			
NEWS	0.806 (0.786 - 0.826)^a^	0.814 (0.808 - 0.821)	0.841 (0.837 - 0.845)
NEWS2	0.816 (0.796 - 0.836)^a^	0.815 (0.808 - 0.821)	0.833 (0.829 - 0.837)
NEWS – NEWS2	−0.010 (-0.023 - 0.003)^a^	0.000 (-0.004 - 0.004)	0.008 (0.007 - 0.010)†

Cardiac arrest			
NEWS	0.701 (0.654 - 0.749)^a^	0.756 (0.744 - 0.769)	0.785 (0.776 - 0.794)
NEWS2	0.706 (0.658 - 0.753)^a^	0.741 (0.728 - 0.754)	0.768 (0.760 - 0.777)
NEWS – NEWS2	−0.004 (-0.046 - 0.037)^a^	0.015 (0.008 - 0.022)†	0.016 (0.012 - 0.020)†

Composite outcome			
NEWS	0.835 (0.824 - 0.847)	0.858 (0.855 - 0.861)	0.881 (0.879 - 0.884)
NEWS2	0.830 (0.818 - 0.841)	0.843 (0.840 - 0.847)	0.867 (0.864 - 0.869)
NEWS – NEWS2	0.006 (-0.003 - 0.014)	0.015 (0.013 - 0.016)†	0.015 (0.014 - 0.016)†

a Where number of adverse outcomes is under 100.

† Denotes significant difference in AUROC (p < 0.05).

Results of the sub-analyses by institution are shown in appendix A3. The effects of using multiple imputation to replace missing vital sign values are shown in appendix A4.

In patients with documented T2RF, the AUROCs for predicting inpatient mortality within 24 h for the two scoring systems were as follows: NEWS 0.862 (95% CI: 0.848 to 0.875); NEWS2 0.841 (0.827 to 0.855) (Table 2). Using a threshold of 5 points, positive predictive values for NEWS and NEWS2 were 2.5% and 3.0% respectively. In patients at risk of T2RF, the AUROC for predicting inpatient mortality within 24 h for the two scoring systems were as follows: NEWS 0.881 (0.878 to 0.884); NEWS2 0.860 (0.857 to 0.864). Using a threshold of 5 points, positive predictive values for NEWS and NEWS2 were 3.2% and 2.7%, respectively.

Our sub-analysis using multiple imputation to deal with missing values gave similar results (appendix A4).

We calculated efficiency curves (see appendix A2) to compare the efficiency of NEWS and NEWS2. The curves demonstrate that, for the few patients with documented T2RF, the use of NEWS2 at the suggested RCPL cut-offs of 5 and 7 points^12^ reduces absolute staff workload by approximately 11% and 5% respectively, but at the expense of reduced sensitivity of approximately 10% and 14%, respectively. For patients at risk of T2RF, the use of NEWS2 at the suggested RCPL cut-offs of 5 and 7 points^13^ does not significantly decrease staff workload, but reduces sensitivity by 5–6%. Finally, if used in error for patients not at risk of T2RF at the suggested RCPL cut-offs, NEWS2 is slightly more sensitive than NEWS but, to achieve this, risks doubling the workload.

## Discussion

### Main findings

This is the first study to evaluate the performance of NEWS2 in hospitalised patients who have documented T2RF or are at risk of it. For the primary outcome - in-hospital death within 24 h of an observation – NEWS2 demonstrated no improvement in discrimination over NEWS for patients with documented T2RF, but at the suggested RCPL cut-offs of 5 and 7 points, the positive predictive values (PPV) were higher for NEWS2 than NEWS. However, for patients at risk of T2RF, NEWS had superior discrimination and higher PPV compared to NEWS2. When applied to patients not at risk of T2RF (to simulate the impact of using NEWS2 in error in such patients) NEWS2 discriminated less well than NEWS and had lower PPV. Finally, NEWS2 did not improve discrimination for any of the secondary outcomes compared to NEWS.

Modified scores have been suggested to account for chronically altered physiology in patients with respiratory-related conditions^10^, ^11^, ^12^. One of these, CREWS^11^, improved the positive predictive value compared to NEWS in patients with or at risk of T2RF (see appendix A5), but at the expense of decreasing sensitivity for events. However, such approaches challenge the premise that a universal EWS, with its attendant advantages, should be employed throughout hospitals. In NEWS2, assigning lower SpO_2_ thresholds together with heuristic weights for the use of supplemental oxygen at higher SpO_2_ values reflects the concern of hyperoxia-induced hypercapnic respiratory failure. However, encoding this concern as undertaken in NEWS2 does not improve discrimination in any of the three risk groups of admissions. Given the main purpose of EWS systems is to identify ill or deteriorating patients, the reduced sensitivity introduced by NEWS2 in patients with documented T2RF and those at risk of it is a disadvantage compared to NEWS. This reduced sensitivity could be ameliorated to an extent by reducing the trigger values for NEWS2, but this would increase staff workload, whilst also introducing further complexity.

The performance of NEWS in this study is similar to that of the original derivation study for NEWS (AUROC, 0.89)^22^ supporting previous external evaluations of the scoring system^32^, ^33^ (see appendix A3 in Supplementary material, which describes the results considering admissions to each trust, separately).

### Strengths

This study focuses on the patient groups for which the new SpO_2_ scoring “scale” in NEWS2 were intended. Robust electronic data capture allowed us to identify groups of patients admitted with/at risk of T2RF; this has not previously been undertaken. Unlike previous studies^32^, our study includes vital signs taken throughout the patient’s hospital journey. The additional analyses, and the TRIPOD statement that guides our work further strengthen the findings of our study, promoting both clarity and interpretability.

### Limitations

Our study relies on diagnostic codes and records of oxygen prescription to categorise patients with/at risk of T2RF, so patients could have been missed or misclassified. However, diagnostic coding for COPD has been shown to be relatively reliable^34^, suggesting using this approach to identify those at risk of T2RF may also be reliable. In the case of oxygen prescriptions, the prescribing clinician’s assessment of whether or not the patient is a “carbon dioxide retainer” is recorded, and it seems likely that the same assessment would underlie the choice of SpO_2_ scale used. Our database does not include documentation of “new confusion”, which is now recommended to be part of the assessment of consciousness on for NEWS2^12^; hence, we could not take account of this in our analysis. Nevertheless, as new confusion was not part of NEWS, our study clearly demonstrates the effect of the differences in oxygen SpO_2_ scales between the two systems for patients with T2RF. Moreover, the absence of this component is unlikely to have a different effect in the risk groups. By analysing each vital sign set as independently associated with outcome (allowing comparison with previous NEWS publications^22^, ^24^) we run the risk of over-representation of some patient groups. However, previous work^35^ suggests allowing an outcome to be represented only once has little effect on assessed outcomes. Evaluation of the secondary outcomes (cardiac arrest and unanticipated ICU admission) in the documented T2RF group should be interpreted with caution given the small number of outcomes (<100).

### Implications

We could find no performance benefit of NEWS2 in any diagnostic group compared to NEWS. If used in error in patients not at risk of T2RF, NEWS2 generally reduces discrimination compared to NEWS. Using NEWS2 instead of NEWS for patients with or at risk of T2RF reduces sensitivity for detecting patients with adverse outcomes. Improving sensitivity could be achieved by reducing the trigger values for NEWS2, but this would also increase staff workload.

The recent endorsement by the RCPL and NHS England of the use of NEWS2 without underpinning evidence makes our study both important and urgent. Implementing NEWS2 requires additional staff training, and new multi-coloured charts, both of which are likely to be costly. The clinical impact of introducing NEWS2 may also have unexpected clinical consequences, some of which may also have financial impact.

Applying the same “normal range” to patients with chronically abnormal physiology (e.g. COPD or heart failure) is a compelling criticism of using a single early warning score (EWS). It is certainly at odds with the interpretation of individual vital signs in clinical practice. However, this possible advantage needs to be counter-balanced with the simplicity of a single system. Applying different scores also creates a more complex protocol and observation chart, potentially increasing staff workload^36^, ^37^. Ultimately, increasing score complexity has to be shown to improve performance for it to be worthwhile.

Our study shows that the modifications made to NEWS2 (specifically, the alternative SpO_2_ scale), which increase chart complexity, are not likely to improve the detection of deterioration and/or reduce false alarms in patients with chronic respiratory disease.

## Conclusion

For patients at risk of, or with documented, T2RF, the changes proposed in NEWS2 do not improve the detection of adverse outcomes, including in-hospital death, unanticipated ICU admission, and cardiac arrest. The intent to account for known physiological differences in patients with chronic respiratory failure is laudable, as are the recommended improvements in the chart for recording oxygen prescriptions. However, the relationship between SpO_2_ values, oxygen therapy and the risk of adverse outcomes should be studied further before wide-scale adoption of NEWS2. In the interim, a more appropriate alternative to changing the weighting system for NEWS, might be to modify the clinical care escalation protocol and response to triggering^38^.

## Funding

This publication presents independent research commissioned by the Health Innovation Challenge Fund (HICF-R9-524; WT-103703/Z/14/Z), a parallel funding partnership between the Department of Health and Wellcome Trust. The views expressed in this publication are those of the authors and not necessarily those of the Department of Health or Wellcome Trust. PJW is supported by the National Institute for Health Research (NIHR), Biomedical Research Centre, Oxford. SG is funded by a NIHR Doctoral Fellowship (DRF-2016-09-073). The views expressed are those of the authors and not necessarily those of the Funders.

## Contributors

Study design: PJW, GSC, SG, JM, PES, GBS, DP; data preparation: OR, MAFP, DP; data analysis: OR, MAFP, DP, SG; data interpretation and writing up of the protocol and paper: all authors contributed.

## Conflicts of interest

VitalPAC™, the system used to collect vital signs data in Portsmouth, is a collaborative development of The Learning Clinic Ltd (TLC) and Portsmouth Hospitals NHS Trust (PHT). At the time of the research, PHT had a royalty agreement with TLC to pay for the use of PHT intellectual property within the VitalPAC™ product. PS is employed by PHT. GS was an employee of PHT until 31/03/2011. DP was an employee of PHT until 31/07/2016. Until October 2015, PS and the wives of GS and DP were minority shareholders in TLC. GS is a member of the Royal College of Physicians of London’s National Early Warning Score (NEWS) Development and Implementation Group (NEWSDIG), which developed NEWS. DP assisted the Royal College of Physicians of London in the analysis of data validating NEWS. PW co-developed the System for Electronic Notification and Documentation (SEND), for which Drayson Health has purchased a sole licence. The company has a research agreement with the University of Oxford and royalty agreements with Oxford University Hospitals NHS Trust and the University of Oxford. Drayson Health may in the future pay PW personal fees.
