# Supplementary material for: A comparison of the ability of the National Early Warning Score and the National Early Warning Score 2 to identify patients at risk of in-hospital mortality: A multi-centre database study
Source: Resuscitation. 2019 Jan;134:147–56. doi: 10.1016/j.resuscitation.2018.09.026 (PMC6995996; doi:10.1016/j.resuscitation.2018.09.026)
Supplement: Supplementary file 1 [file mmc1.docx]

**A comparison of the ability of the National Early Warning Score and the National Early Warning Score 2 to identify patients at risk of in-hospital mortality: a multi-centre database study.**

**Supplemental Material - Appendix**

Marco AF Pimentel, Postdoctoral Researcher, Institute of Biomedical Engineering, Department of Engineering Science, University of Oxford, Oxford, UK

Oliver C Redfern, Research Fellow, Centre for Healthcare Modelling and Informatics, University of Portsmouth, Portsmouth, UK

Stephen Gerry, Medical Statistician and NIHR Doctoral Research Fellow, Centre for Statistics in Medicine, Nuffield Department of Orthopaedics, Rheumatology and Musculoskeletal Sciences, Botnar Research Centre, University of Oxford, Oxford, UK

Gary S Collins, Professor of Medical Statistics, Centre for Statistics in Medicine, Nuffield Department of Orthopaedics, Rheumatology and Musculoskeletal Sciences, Botnar Research Centre, University of Oxford, Oxford, UK

James Malycha, Clinical Research Fellow, Nuffield Department of Clinical Neurosciences, Oxford University Hospitals NHS Trust, Oxford, UK

David Prytherch, Professor of Health Informatics, Centre for Healthcare Modelling and Informatics, University of Portsmouth, Portsmouth, UK

Paul E Schmidt, Consultant Physician in Acute Medicine, Portsmouth Hospitals NHS Trust, Portsmouth, UK

Gary B Smith, Professor, Faculty of Health and Social Sciences, Bournemouth University, Bournemouth, UK

Peter J Watkinson, Associate Professor of Intensive Care Medicine, Nuffield Department of Clinical Neurosciences, Oxford University Hospitals NHS Trust, Oxford, UK

**Correspondence to:**

Marco AF Pimentel,

Postdoctoral Researcher,

Institute of Biomedical Engineering,

Department of Engineering Science,

University of Oxford,

Oxford

UK

Email: marco.pimentel@eng.ox.ac.uk

## APPENDIX

### A1. Scoring systems

Table A1 depicts the weights and cut-offs for the three different scoring systems included in this study: NEWS and NEWS2. The two scores contain the same number of components (vital signs), and only the weights and cut-offs assigned to oxygen saturation (SpO_2_) are. In this study, we did not include the “new confusion” component proposed for NEWS2^12^, as these data were unavailable.


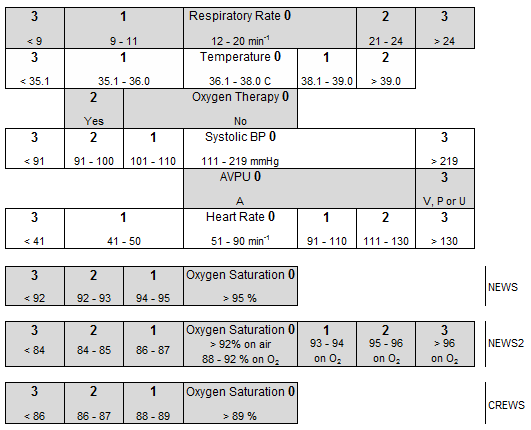


Table A1. NEWS and NEWS2 scoring systems. The weights assigned to respiratory rate, temperature, systolic blood pressure, AVPU, heart rate, and whether the patient is on supplemental oxygen, are the same for the three scoring systems. Only the scoring scale for oxygen saturation (on SpO_2_) is different. We note that for NEWS2, weights given depend on both SpO_2_ and whether the patient is on supplemental oxygen (this corresponds to the SpO_2_ scale 2 of NEWS2, and the SpO_2_ scale 1 is the same as NEWS).

### A2. EWS Efficiency curves

Figure A2 shows the “EWS efficiency curve” for NEWS and NEWS2 using the primary outcome of in-hospital mortality within 24 hours of the observation set.


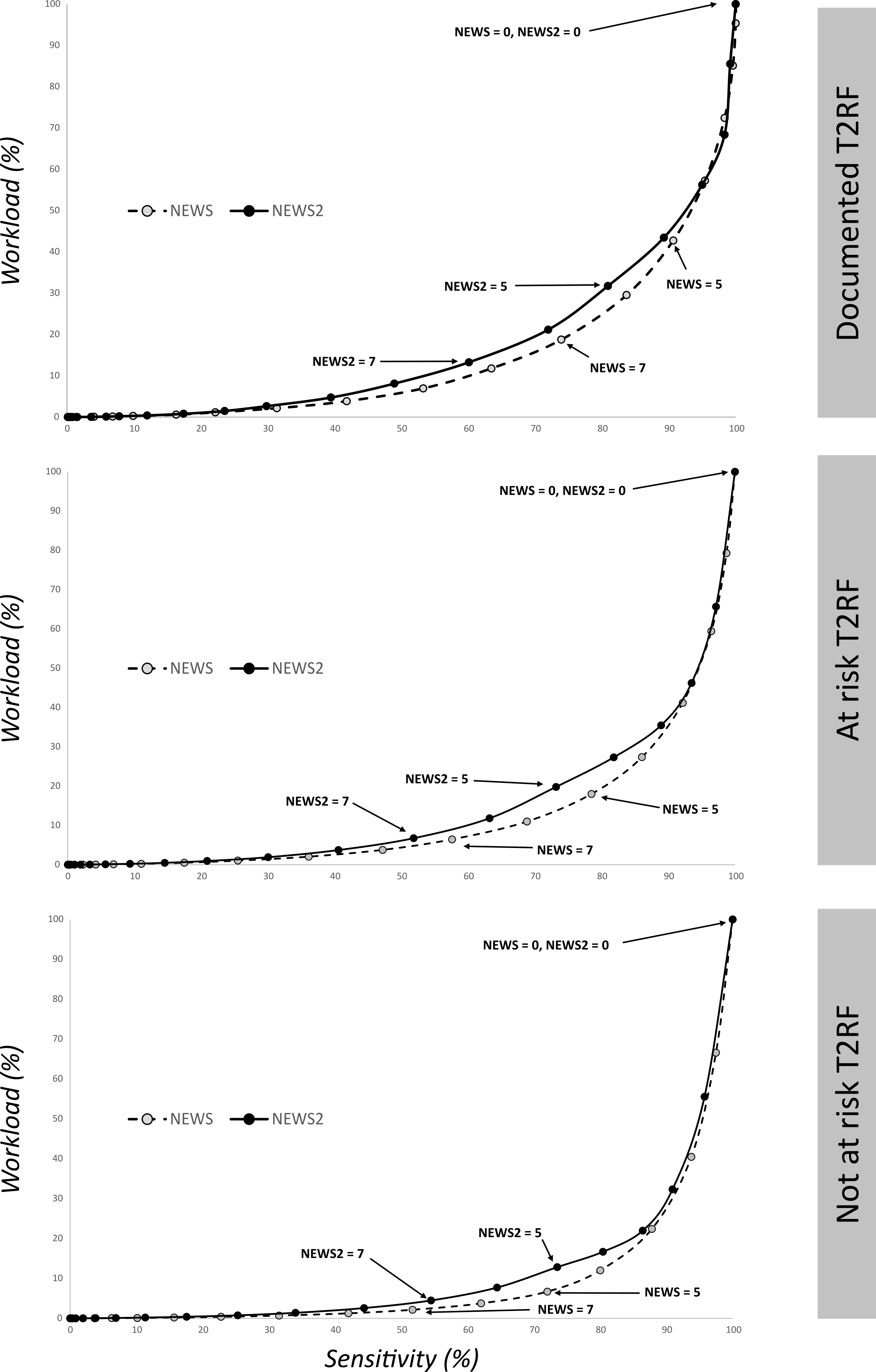


Figure A2. “EWS efficiency curve^21^” for the two scoring systems in each of the three risk groups. Each curve shows the percentage of the total number of observations at, or above, each EWS value against the percentage of the total number of observations for which the primary outcome was true at, or above, that EWS value.

It has been suggested that the “efficiency” metric, introduced in a previous study^21^, provides a relative measure of the number of “triggers” that would be generated at different values of a EWS, and permits the comparison of the workload generated by different EWSs. As an example, in the second risk group (middle plot, in figure A2), a NEWS2 of 5 or above would generate a trigger in 20% of observations, and this would “detect” 73% of observations that were followed by death within the following 24 hours in patients at risk of type II respiratory rate. At the same threshold, in the same risk group, NEWS would trigger a smaller proportion of observations (18%) with a higher sensitivity (78%). We observe that, with this concept, NEWS2 would generate a higher workload and reduce sensitivity.

### A3. Unicentre analysis

Given the potential differences between admissions to both trusts, and the different periods covered, we conducted a sub-analysis, in which we considered admissions to each trust separately. Table A3-1 summarises the number of admissions considered in each risk group, together with their corresponding demographic descriptors and other clinical information.

|  | **Documented T2RF** | **At risk T2RF** |  | **Not at risk of T2RF** | |
| --- | --- | --- | --- | --- | --- |
|  | **OUH** | **OUH** | **PH** | **OUH** | **PH** |
| Admission period | 2016 | 2016 | 2012-2016 | 2016 | 2012-2016 |
| Number of admissions | 1,394 | 7,466 | 41,432 | 39,758 | 162,336 |
| Males, N (%) | 696  (49.9) | 3,814  (51.1) | 19,755  (47.7) | 19,379  (48.7) | 76,359  (47.0) |
| Age (years),  median (IQR) | 75 (67-83) | 72 (61-80) | 72 (60-80) | 65 (47-79) | 66 (47-80) |
| Length of stay (days),  median (IQR) | 6.7 (3.1-14) | 4.1 (1.8-9.3) | 3.9 (1.8-8.9) | 2.8 (1.3-6.5) | 2.8 (1.3-6.8) |
| Charlson Comorbidity Index, median (IQR)* | 7 (4-16) | 6 (4-14) | 4 (0-14) | 0 (0-8) | 0 (0-8) |
| Elective admissions (%) | 104 (7.5) | 1434 (19.2) | 7917 (19.1) | 11532 (29) | 38448 (23.7) |
| Surgical admissions (%) | 229 (16.4) | 2611 (35) | 12222 (29.5) | 20303 (51.1) | 69126 (42.6) |
| Ethnic category (%) |  |  |  |  |  |
| Asian or Asian British | 23 (1.6) | 153 (2) | 128 (0.3) | 915 (2.3) | 935 (0.6) |
| Black or Black British | 1 (0.1) | 46 (0.6) | 70 (0.2) | 393 (1) | 638 (0.4) |
| Mixed | 9 (0.6) | 40 (0.5) | 77 (0.2) | 256 (0.6) | 454 (0.3) |
| Other Ethnic Groups | 126 (9) | 755 (10.1) | 3562 (8.6) | 6938 (17.5) | 22647 (14) |
| Not disclosed | 5 (0.4) | 26 (0.3) | 116 (0.3) | 370 (0.9) | 587 (0.4) |
| White | 1230  (88.2) | 6446  (86.3) | 37479  (90.5) | 30886  (77.7) | 137079 (84.4) |
| Primary outcome, N (%) |  |  |  |  |  |
| In-hospital mortality | 159 (11.4) | 325 (4.4) | 1,895 (4.6) | 733 (1.8) | 3,873 (2.4) |
| Secondary outcome, N (%) |  |  |  |  |  |
| Unanticipated ICU | 45 (3.2) | 114 (1.5) | 461 (1.1) | 436 (1.1) | 1,268 (0.8) |
| Cardiac arrest | 18 (1.3) | 37 (0.5) | 251 (0.6) | 64 (0.2) | 564 (0.3) |
| Number of vital sign sets | 61,340 | 219,468 | 1,246,952 | 910,209 | 3,841,114 |

Table A3-2. Demographic descriptors for admissions included in each risk group, from each trust: Oxford University Hospitals (OUH) and Portsmouth Hospital (PH) NHS Trusts. We note that we were only able to identify Documented T2RF admissions in OUH.

T2RF denotes Type II Respiratory Failure.

*The Charlson Comorbidity Index was determined according to the methodology and specification provided by NHS Digital (available at <https://beta.digital.nhs.uk/publications/ci-hub/summary-hospital-level-mortality-indicator-shmi>).

Within each risk group, we do not observe substantial differences in key demographics and in-hospital mortality between OUH and PH subgroups.

The AUROC (95% CI) values for all scoring systems calculated for each sub-group of admissions using the primary and secondary outcomes are presented in table A3-2. If we consider each trust separately, we observe that the performance of NEWS2 is consistently not superior to NEWS using the different outcomes. For example, in admissions to OUH categorised as being at risk of type II respiratory failure, the AUROCs for predicting in-hospital death for the two scoring systems were as follows: NEWS 0.893 (0.884 – 0.901); NEWS2 0.876 (0.867 – 0.885). Similarly, in the same group of admissions to PH, the AUROCs were: NEWS 0.880 (0.876 – 0.884); NEWS2 0.858 (0.854 – 0.862).

|  | **Documented T2RF** | **At risk T2RF** | | **Not at risk T2RF** | |
| --- | --- | --- | --- | --- | --- |
|  | **OUH** | **OUH** | **PH** | **OUH** | **PH** |
| **In-hospital death** |  |  |  |  |  |
| NEWS | 0.862 (0.848 - 0.875) | 0.893 (0.884 - 0.901) | 0.880 (0.876 - 0.884) | 0.923 (0.917 - 0.928) | 0.909 (0.906 - 0.911) |
| NEWS2 | 0.841 (0.827 - 0.854) | 0.876 (0.867 - 0.885) | 0.858 (0.854 - 0.862) | 0.902 (0.896 - 0.908) | 0.891 (0.888 - 0.893) |
| **Unanticipated ICU** |  |  |  |  |  |
| NEWS | 0.806* (0.786 - 0.826) | 0.843 (0.828 - 0.858) | 0.807 (0.799 - 0.815) | 0.831 (0.823 - 0.840) | 0.843 (0.838 - 0.847) |
| NEWS2 | 0.816* (0.796 - 0.836) | 0.839 (0.824 - 0.853) | 0.808 (0.800 - 0.816) | 0.826 (0.817 - 0.835) | 0.833 (0.829 - 0.838) |
| **Cardiac arrest** |  |  |  |  |  |
| NEWS | 0.701* (0.654 - 0.749) | 0.767* (0.734 - 0.801) | 0.756 (0.742 - 0.769) | 0.783* (0.756 - 0.811) | 0.788 (0.779 - 0.798) |
| NEWS2 | 0.706* (0.658 - 0.753) | 0.764* (0.731 - 0.798) | 0.738 (0.724 - 0.752) | 0.760* (0.732 - 0.788) | 0.773 (0.764 - 0.782) |
| **Composite outcome** |  |  |  |  |  |
| NEWS | 0.835 (0.824 - 0.847) | 0.869 (0.861 - 0.877) | 0.857 (0.853 - 0.860) | 0.875 (0.870 - 0.881) | 0.883 (0.881 - 0.886) |
| NEWS2 | 0.830 (0.818 - 0.841) | 0.858 (0.850 - 0.866) | 0.841 (0.837 - 0.844) | 0.862 (0.856 - 0.867) | 0.868 (0.866 - 0.871) |

Table A3-2. Performance metrics of the two scoring systems (NEWS and NEWS2) for predicting the primary (in-hospital death) and secondary outcomes in each of the sub-groups: area under the receiver operating characteristics curve (AUROC), and 95% confidence interval (CI).

T2RF denotes Type II Respiratory Failure.

* Where number of adverse outcomes is under 100 (as per table A3-1).

### A4. Multiple imputation for dealing with missing data

The performance of each scoring system (NEWS and NEWS2) was evaluated using multiple imputation. All observations sets from OUH were used for this analysis (representing all three risk groups). The proportion of missing data for each variable is shown in table A4-1a. Temperature is the vital sign most often not recorded in OUH (9.3% of observation sets do not include a recorded temperature). Summary value for observation sets with and without missing data are shown in table A4-1b.

| Vital sign | Number of missing observations | % Total observations |
| --- | --- | --- |
| Temperature | 124,372 | 9.3 |
| Heart rate | 26,930 | 2.0 |
| Systolic blood pressure | 24,978 | 1.9 |
| AVPU | 61,099 | 4.5 |
| Oxygen saturations | 27,344 | 2.0 |
| On supplementary oxygen | 49,688 | 3.7 |
| Respiratory rate | 41,821 | 3.1 |

Table A4-1a. Proportion of missing data for each vital sign in the OUH data set. A total of 1,345,040 observation sets are included in the analysis.

|  | **Observation sets**  **with missing data** | **Complete**  **observation sets** |
| --- | --- | --- |
| Age (years) | 70 (56-81) | 71 (55-82) |
| AVPU (% Alert) | 63.6% | 97.8% |
| Charlson Co-morbidity Index (CCI) | 4 (0-13) | 4 (0-14) |
| Heart rate | 78 (67-90) | 80 (70-91) |
| Males (%) | 50% | 50.1% |
| On oxygen therapy (%) | 33.1% | 18.8% |
| Respiratory rate | 17 (15-18) | 17 (16-18) |
| Systolic blood pressure | 123 (108-140) | 125 (111-141) |
| Peripheral oxygen saturations | 97 (95-99) | 96 (95-98) |
| Temperature | 36.4 (36-37) | 36.4 (36-37) |

Table A4-1b. Vital sign and demographic descriptors for observation sets with and without missing data in the OUH data set. The median and interquartile range is given for each variable, unless otherwise specified.

We conducted this analysis using the Amelia II^[[1]](#footnote-2)^ package, for the R statistical software (v3.4.4).

Twenty data sets with imputed data were created, and for each permutation of risk group and outcome, AUROC values and their standard errors were calculated and combined using Rubin’s rule^[[2]](#footnote-3)^.

Table A4-2 shows the performance of the three systems in the three risk groups (we also compare the performance in same risk groups using only complete sets of observations, as previously shown in the section A3). We note that the results using multiple imputation are comparable to those using only complete sets of observations.

|  | **Documented T2RF** | | **At risk T2RF** | | **Not at risk T2RF** | |
| --- | --- | --- | --- | --- | --- | --- |
|  | **Complete sets** | **Mult. Imp.** | **Complete sets** | **Mult. Imp.** | **Complete sets** | **Mult. Imp.** |
| **In-hospital death** |  |  |  |  |  |  |
| NEWS | 0.862  (0.848 - 0.875) | 0.866  (0.853 - 0.877) | 0.893  (0.884 - 0.901) | 0.899  (0.891 - 0.906) | 0.923  (0.917 - 0.928) | 0.924  (0.919 - 0.929) |
| NEWS2 | 0.841  (0.827 - 0.855) | 0.844  (0.832 - 0.856) | 0.876  (0.867 - 0.885) | 0.876  (0.868 - 0.883) | 0.902 (0.896 - 0.908) | 0.896  (0.891 - 0.901) |
|  |  |  |  |  |  |  |
| **Unanticipated ICU** |  |  |  |  |  |  |
| NEWS | 0.806* (0.786 - 0.826) | 0.796* (0.777 - 0.815) | 0.843 (0.828 - 0.858) | 0.836  (0.822 - 0.848) | 0.831 (0.823 - 0.840) | 0.828  (0.820 - 0.835) |
| NEWS2 | 0.816* (0.796 - 0.836) | 0.817* (0.799 - 0.834) | 0.839 (0.824 - 0.853) | 0.836  (0.822 - 0.848) | 0.826 (0.817 - 0.835) | 0.821  (0.813 0.828) |
|  |  |  |  |  |  |  |
| **Cardiac arrest** |  |  |  |  |  |  |
| NEWS | 0.701* (0.654 - 0.749) | 0.683*  (0.635 - 0.728) | 0.767 (0.734 - 0.801) | 0.766  (0.734 - 0.796) | 0.783* (0.756 - 0.811) | 0.792  (0.766 - 0.816) |
| NEWS2 | 0.706* (0.658 - 0.753) | 0.689*  (0.637- 0.736) | 0.764 (0.731 - 0.798) | 0.759  (0.726- 0.789) | 0.760* (0.732 - 0.788) | 0.755  (0.731 - 0.778) |
|  |  |  |  |  |  |  |
| **Composite outcome** |  |  |  |  |  |  |
| NEWS | 0.835 (0.824 - 0.847) | 0.835  (0.824 - 0.845) | 0.869  (0.861 - 0.877) | 0.870  (0.863 - 0.877) | 0.875 (0.870 - 0.881) | 0.874  (0.869 - 0.878) |
| NEWS2 | 0.830 (0.818 - 0.841) | 0.833  (0.822 - 0.843) | 0.858  (0.850 - 0.866) | 0.857  (0.849 - 0.864) | 0.862 (0.856 - 0.867) | 0.856  (0.852 - 0.861) |
|  |  |  |  |  |  |  |

Table A4-2. Performance metrics of the two scoring systems (NEWS and NEWS2) for predicting the primary (in-hospital death) and secondary outcomes using (1) all vital-sign observation sets and multiple imputation (“Mult. Imp.”) for dealing with missing data, and (2) only complete vital-sign observation sets. Area under the receiver operating characteristics curve (AUROC), and 95% confidence interval (CI) are shown.

T2RF denotes Type II Respiratory Failure.

* Where number of adverse outcomes is under 100 (as per table A3-1).

### A5. Evaluation of CREWS

Table A5-1 shows the discrimination of NEWS and NEWS2 in each hospital trust (as also presented in Table A3-2), compared with the CREWS system. Table A5-2 shows the difference in AUROC values for NEWS2 and CREWS, relative to NEWS. Confidence intervals and p-values were calculated using bootstrapping with 2000 replicates^[[3]](#footnote-4)^.

|  | **Documented T2RF** | **At risk T2RF** | | **Not at risk T2RF** | |
| --- | --- | --- | --- | --- | --- |
|  | **OUH** | **OUH** | **PH** | **OUH** | **PH** |
| **In-hospital death** |  |  |  |  |  |
| NEWS | 0.862 (0.848 - 0.875) | 0.893 (0.884 - 0.901) | 0.880 (0.876 - 0.884) | 0.923 (0.917 - 0.928) | 0.909 (0.906 - 0.911) |
| NEWS2 | 0.841 (0.827 - 0.854) | 0.876 (0.867 - 0.884) | 0.858 (0.854 - 0.862) | 0.902 (0.896 - 0.908) | 0.891 (0.888 - 0.893) |
| CREWS | 0.864 (0.851 - 0.878) | 0.895 (0.886 - 0.903) | 0.881 (0.877 - 0.884) | 0.917 (0.911 - 0.922) | 0.902 (0.900 - 0.905) |
| **Unanticipated ICU** |  |  |  |  |  |
| NEWS | 0.806* (0.786 - 0.826) | 0.843 (0.828 - 0.858) | 0.807 (0.799 - 0.815) | 0.831 (0.823 - 0.840) | 0.843 (0.838 - 0.847) |
| NEWS2 | 0.816* (0.796 - 0.836) | 0.839 (0.824 - 0.853) | 0.808 (0.800 - 0.816) | 0.826 (0.817 - 0.835) | 0.833 (0.829 - 0.838) |
| CREWS | 0.819* (0.799 - 0.839) | 0.846 (0.832 - 0.861) | 0.816 (0.808 - 0.824) | 0.832 (0.823 - 0.841) | 0.841 (0.836 - 0.845) |
| **Cardiac arrest** |  |  |  |  |  |
| NEWS | 0.701* (0.654 - 0.749) | 0.767* (0.734 - 0.801) | 0.756 (0.742 - 0.769) | 0.783* (0.756 - 0.811) | 0.788 (0.779 - 0.798) |
| NEWS2 | 0.706* (0.658 - 0.753) | 0.764* (0.731 - 0.798) | 0.738 (0.724 - 0.752) | 0.760* (0.732 - 0.788) | 0.773 (0.764 - 0.782) |
| CREWS | 0.716* (0.669 - 0.763) | 0.777* (0.744 - 0.810) | 0.758 (0.744 - 0.772) | 0.777* (0.749 - 0.804) | 0.780 (0.771 - 0.790) |
| **Composite outcome** |  |  |  |  |  |
| NEWS | 0.835 (0.824 - 0.847) | 0.869 (0.861 - 0.877) | 0.857 (0.853 - 0.860) | 0.875 (0.870 - 0.881) | 0.883 (0.881 - 0.886) |
| NEWS2 | 0.830 (0.818 - 0.841) | 0.858 (0.850 - 0.866) | 0.841 (0.837 - 0.844) | 0.862 (0.856 - 0.867) | 0.868 (0.866 - 0.871) |
| CREWS | 0.844 (0.832 - 0.855) | 0.872 (0.865 - 0.880) | 0.859 (0.856 - 0.863) | 0.872 (0.867 - 0.878) | 0.878 (0.876 - 0.881) |

Table A5-1. Performance metrics of the two scoring systems (NEWS, NEWS2) compared with CREWS for predicting the primary (in-hospital death) and secondary outcomes in each of the sub-groups: area under the receiver operating characteristics curve (AUROC), and 95% confidence interval (CI).

T2RF denotes Type II Respiratory Failure.

* Where number of adverse outcomes is under 100 (as per table A3-1).

|  | **Documented T2RF** | **At risk T2RF** | | **Not at risk T2RF** | |
| --- | --- | --- | --- | --- | --- |
|  | **OUH** | **OUH** | **PH** | **OUH** | **PH** |
| **In-hospital death** |  |  |  |  |  |
| NEWS2 | 0.021 (0.012 - 0.030)^†^ | 0.017 (0.012 - 0.022)^†^ | 0.022 (0.020 - 0.024)^†^ | 0.021 (0.018 - 0.023)^†^ | 0.018 (0.017 - 0.019)^†^ |
| CREWS | -0.003 (-0.008 - 0.003) | -0.002 (-0.005 - 0.001) | 0.000 (-0.002 - 0.001) | 0.006 (0.004 - 0.008)^†^ | 0.007 (0.006 - 0.007)^†^ |
| **Unanticipated ICU** |  |  |  |  |  |
| NEWS2 | -0.010* (-0.023 - 0.003) | 0.004 (-0.003 - 0.012) | -0.001 (-0.005 - 0.003) | 0.006 (0.002 - 0.009)^†^ | 0.009 (0.007 - 0.011)^†^ |
| CREWS | -0.014* (-0.022 - -0.006)^†^ | -0.003 (-0.009 - 0.002) | -0.009 (-0.012 - -0.006)^†^ | 0.000 (-0.003 - 0.002) | 0.002 (0.000 - 0.004)^†^ |
| **Cardiac arrest** |  |  |  |  |  |
| NEWS2 | -0.004* (-0.046 - 0.037) | 0.003* (-0.017 - 0.023) | 0.017 (0.010 - 0.025)^†^ | 0.023* (0.013 - 0.033)^†^ | 0.015 (0.011 - 0.020)^†^ |
| CREWS | -0.015* (-0.037 - 0.008) | -0.010* (-0.024 - 0.004) | -0.002 (-0.007 - 0.003) | 0.007* (0.002 - 0.015) | 0.008 (0.004 - 0.011)^†^ |
| **Composite outcome** |  |  |  |  |  |
| NEWS2 | 0.006 (-0.003 - 0.014) | 0.011 (0.006 - 0.015)^†^ | 0.016 (0.014 - 0.018)^†^ | 0.013 (0.011 - 0.015)^†^ | 0.015 (0.014 - 0.016)^†^ |
| CREWS | -0.008 (-0.013 - -0.004)^†^ | -0.003 (-0.006 - -0.001)^†^ | -0.003 (-0.004 - -0.001)^†^ | 0.003 (0.001 - 0.004)^†^ | 0.005 (0.004 - 0.006)^†^ |

Table A5-2. Differences in AUROC values of the NEWS2 and CREWS, relative to NEWS for predicting the primary (in-hospital death) and secondary outcomes in each of the sub-groups.

T2RF denotes Type II Respiratory Failure.

*Where number of adverse outcomes is under 100 (as per table A3-1).

^†^ Significant difference in AUROC (p < 0.05).

1. Amelia II: A Program for Missing Data. Journal of Statistical Software 45(7):1-47. [↑](#footnote-ref-2)
2. Rubin, D. Multiple Imputation for Nonresponse in Surveys. New York: Wiley 1987. [↑](#footnote-ref-3)
3. Robin X, Turck N, Hainard A, Tiberti N, Lisacek F, Sanchez J-C, et al. pROC: an open-source package for R and S+ to analyze and compare ROC curves. BMC Bioinformatics 2011;12:77. [↑](#footnote-ref-4)
